# The Hampstead Appointment

**Published:** 1915-02-06

**Authors:** 


### THE EDITOR'S LETTER-BOX.

### The Hampstead Appointment.

*To the Editor of* The Hospital.

Sir,—With reference to your notes about the appointment of a secretary to the Hampstead General Hospital, three points have occurred to me about which I should be glad of your opinion and that of your readers.

The advertisement of the appointment states as an express condition: "Candidates *must* have had previous hospital experience." Therefore, in appointing a gentleman whom you say "appears to have had no experience of this kind," did not the Council commit: (1) A breach of faith with applicants who were actual hospital officers and (2) a breach of faith with the public, who have a right to expect a man of the best obtainable experience to be appointed; and (3) is the appointment, in contravention of an advertised condition, valid? Yours faithfully,

Interested.

January 30, 1915.

[The answer to our correspondent's questions appears to be as follows:—(1) and (3) The author of an advertisement is not compelled to bind himself by his own provisions; (2) the Council are the trustees of the public, which, save with grave reason, must assume them to be acting in the interests of the confidence reposed in them, and this includes the exercise of their discretion, subject to responsibility for their actions.—Ed. The Hospital.]

